# Editorial: Multi-dimensional characterization of neuropsychiatric disorders

**DOI:** 10.3389/fnins.2022.1089886

**Published:** 2022-12-01

**Authors:** Peng Wang, Shijie Zhao, Xiang Li, Jinglei Lv

**Affiliations:** ^1^Fuwai Hospital, Chinese Academy of Medical Sciences and Peking Union Medical College, Beijing, China; ^2^School of Automation, Northwestern Polytechnical University, Xi'an, China; ^3^Research and Development Institute of Northwestern Polytechnical University in Shenzhen, Shenzhen, China; ^4^Center for Advanced Medical Computing and Analysis (CAMCA), Department of Radiology, Massachusetts General Hospital and Harvard Medical School, Boston, MA, United States; ^5^School of Biomedical Engineering and Brain and Mind Center, University of Sydney, Sydney, NSW, Australia

**Keywords:** neuropsychiatric disorders (NPD), NeuroImage, electroencephalography, PET, MRI

## Introduction

The human nervous system itself and its extensive connections with the human body form a complex system. The individual characteristics and abnormalities of the system can be observed on multiple dimensions, such as the brain functional, structural, molecular, genetic, and behavioral dimensions. Understanding the underlying multi-dimensional mechanisms and the corresponding biomarkers for neuropsychiatric disorders could be regarded as the highest-priority goal in neuroscience and a vital step for clinical practice (Li et al., 2019). In particular, brain disorders can be characterized on multiple dimensions, with reference either to the biomarkers of one modality or to those of multiple modalities. This multi-dimensional method of characterization provides a more objective and accurate identification of disorders; based on this level of precision, treatments can be developed to benefit patients in clinical practice.

This Research Topic assembles 10 articles on a broad spectrum of research in neuropsychiatric disorders. Authors from backgrounds in psychiatry, radiology, computer science, and engineering have all contributed to this Research Topic by conducting empirical studies, developing computational models, performing reviews, and introducing novel intervention techniques. In this Editorial, we provide an overview of these exciting and diverse articles, grouping them based on their conceptual design.

## Computational modeling of multi-dimensional brain signals

The modeling scheme proposed by Lun et al. extracts features of motor imagery EEG signals separately for each hemisphere using a deep learning architecture and then combines the embeddings for classification. This research is helpful for the development of brain–machine interfaces for motor functional disability and spinal cord injury. The article by Peng et al. proposes to characterize the neural electrical signals captured by intracranial and scalp EEG on multiple temporal and frequency dimensions so that the rich information can be better utilized for seizure prediction in epilepsy. This is particularly important in surgery-planning for drug-resistant epilepsy. The model proposed by Liu K. et al. utilizes coupled integration for hierarchical feature representation of MCI and AD with structural MRI to enable improved discrimination of the different stages of AD. Also targeting AD diagnosis but using PET imaging, the article by Cui et al. proposes a region-by-region descriptor for FDG-PET. The collective descriptors are fed into a novel deep learning network (BMNet) featuring bilinear pooling and metric learning. It transpires that this method offers improved performance in the identification of EMCI and LMCI. This work is important for early diagnosis and intervention in AD.

## Multi-dimensional data for treatment development

Sun, Guo et al. used an fMRI-derived measure of ALFF to identify potential age differences in the neuropathological mechanism of treatment-resistant depression. An investigation by Shadli et al. tested the possibility of using EEG as a biomarker for ketamine therapy in anxiety disorders. Among the signals from multiple electrodes and the frequency spectrum, the authors report right frontal theta power to be a possible biomarker. Wu et al. combined rs-fMRI, protein markers, and behavioral assessments to investigate the effect of rTMS on neural plasticity. Although this is a pre-clinical study, it provides evidence for cognitive enhancement that may have future human applications.

## Identification and validation of imaging biomarkers of neuropsychiatric disorders

In a study of structural imaging biomarkers, Liu T. et al. investigated multiple variables relating to the structural connectome, measured with diffusion MRI. They found that local efficiency of the structural connectome is correlated with language function in infants, suggesting a relationship between language disorders and the early development of white matter in infancy. Functional brain signals also provide an effective approach to the characterization of brain alterations and treatment response. Under the approach proposed by Sun, Chen et al., the rich information obtained though rs-fMRI is modeled in the form of regional ReHo and ALFF, resulting in the discovery of functional alteration imaging biomarkers for first-episode and recurrent depression; this finding provides neuroimaging insights into the psychological mechanism of depression. Last but not least, the review article by Pan et al. revisits the efforts of the psychiatric and neuroimaging community over the course of 40 years to understand the neural substrates of post-stroke depression, covering regional lesion analysis and the study of brain networks, from structural to functional connectome. It is emphasized in this review that multivariate analysis has played an important role in the task, thereby further highlighting the importance of multi-dimensional characterization of the disorder.

In the current Research Topic, most of the articles have devoted efforts to the characterization of neuropsychiatric disorders within a single modality. We envision that, in the near future, it will be possible to measure many of these multi-dimensional modalities in a single patient, so that information across modalities can be integrated to provide a more comprehensive characterization of a particular disorder or spectrum of disorders. Such high-dimensional multi-modal characterization would provide higher discriminability and more accurate digital identification. High-dimensional characterization will become even more powerful if this method can be employed with a large-scale patient cohort, as the big data generated in this way can be fed into machine learning and artificial intelligence systems, enabling an improved understanding of the mechanisms of the disorder in question. As a consequence, we will be able to characterize patient subtypes more precisely and provide personalized diagnoses, resulting in improved treatment and early intervention for neuropsychiatric disorders.

## Author contributions

PW: writing—original draft. SZ, XL, and JL: writing—review and editing. All authors contributed to the article and approved the submitted version.

## Funding

This work was supported in part by the National Key R&D Program of China under Grant Number: 2020AAA0105702 and the National Natural Science Foundation of China under Grant Numbers: 42271315, 61936007, 82060336, 62036011, and U1801265. Guangdong Basic and Applied Basic Research Foundation (2214050008706), Science and Technology Support Project of Guizhou Province under Grant Number: [2021]432, and Shenzhen Science and Technology Program (JCYJ20220530161409021).

## Conflict of interest

The authors declare that the research was conducted in the absence of any commercial or financial relationships that could be construed as a potential conflict of interest.

## Publisher's note

All claims expressed in this article are solely those of the authors and do not necessarily represent those of their affiliated organizations, or those of the publisher, the editors and the reviewers. Any product that may be evaluated in this article, or claim that may be made by its manufacturer, is not guaranteed or endorsed by the publisher.
